# The complete mitochondrial genome of *Bullanga florida* (Neuroptera: Myrmeleontidae)

**DOI:** 10.1080/23802359.2016.1214548

**Published:** 2016-09-05

**Authors:** Xu-E Lan, Shan Chen, Fen-Hong Li, Ping You

**Affiliations:** College of Life Science, Shaanxi Normal University, Xi’an, China

**Keywords:** Mitochondrial genome, *Bullanga florida*, Neuroptera, phylogenetic

## Abstract

The complete mitochondrial genome (mitogenome) of *Bullanga florida* (Neuroptera: Myrmeleontidae) was determined. The entire sequence is 15,937 bp in length which contains 13 protein-coding genes (PCGs), 22 tRNAs, two rRNAs and one non-coding AT-rich region. The overall A + T content of mitogenome is 73.3%. The gene order and arrangement are similar to other Neuroptera mitogenomes. Thirteen PCGs start with standard ATN initiation codons and stop with termination codons TAA or T. All tRNA genes have a typical clover-leaf secondary structure except for *tRNA^Ser^* (AGN), whose dihydrouridine (DHU) arm do not form a stable stem-loop structure. Two rRNA genes (*rrnL* and *rrnS*) are 1325 bp and 776 bp in size, respectively. The phylogenetic analysis is based on the amino acid sequences of 13 PCGs indicates that *B. florida* is a sister group to *Epacanthaclisis banks*, and Myrmeleontiformia is the monophyletic group.

Neuroptera is one of the oldest holometabolous surviving insects, approximately 6000 species have been described so far. Myrmeleontidae is the most diverse and widely distributed. There were more than 2000 species has been reported in the world (Huang et al. 2008; Zhang et al. 2014). The larvae antlion and adult generally prey pests as ecological beneficial insects. At present, only two complete mitogenome sequences have been published from Myrmeleontidae in GenBank database.

The adults of *Bullanga florida* (Navás, 1913) were collected from the Xunyangba (33.33°N, 108.33°E), Ningshan County, Shaanxi Province in the Qinling Mountain region of central China. DNA is extracted from adult specimen. The mitogenome of *B. florida* was sequenced after PCR amplification, sequencing and annotations. Voucher specimens have been deposited in the Insect Collection (Accession Number SNU-Neu-20150001-5), College of Life Sciences, Shaanxi Normal University, Xi’an, China 710062.

The complete mitogenome is a typical circular DNA molecule with 15,937 bp in length (GenBank accession number KX369241) which consists of a set of 37 genes (13 PCGs, 22 tRNA genes and two rRNA genes) and one major non-coding AT-rich region. The gene order and arrangement are identical to other Neuroptera species (Beutel et al. 2010; Negrisolo et al. 2011; Cheng et al. 2014; Yan et al. 2014). The H chain codes 23 genes, and other 14 genes are coded by the L chain. The mitogenome has a total 138 bp intergenic spacer sequences with the longest 57 bp located between *tRNA^Ile^* and *tRNA^Gln^*. The complete mitogenome has a sensible AT bias with A + T content of 73.3%. The overall base composition of *B. florida* mitogenome is: A (38.8%), T (34.5%), C (15.8%), G (10.9%).

The total nucleotide length of the protein-coding genes is 11,176 bp with 71.2% A + T content. All PCGs use ATN (ATA, ATT, ATG, ATC) as start codon. Nine genes terminate with TAA or TAG as stop codon. The remaining four genes (*COI*, *COII*, *ND5* and *ND4*) have an incomplete stop codon T. Large ribosomal subunit (*rrnL*) gene is located between *tRNA^Leu^*(CUN) and *tRNA^Val^* which has a length of 1325 bp, and the 776 bp small ribosomal subunit (*rrnS*) gene is located between *tRNA^Val^* and the A + T-rich region. The total length of tRNA is 1464 bp with 75.1% A + T content. All 22 tRNA ranging from 64 to 72 bp have typical cloverleaf secondary structure except for *tRNA^Ser^*(AGN), which dihydrouridine(DHU) is absent and this common construction generally exists in metazoan mitogenomes (Ye et al. 2014). Located between *rrnS* and the *tRNA^Ile^*-*tRNA^Gln^*-*tRNA^Met^* gene cluster is non-coding A + T-rich region with 1066 bp in length and A + T content of 85.0%, including a 10 bp poly-T stretch.

In order to investigate the phylogenetic status of *B. florida*, two methods (Bayesian inference (BI) and Maximum likelihood (ML)) were used for phylogenetic reconstruction (Figure 1). The BI analyses and the ML analyses of 13 PCGs dataset showed the similar topology. *Bullanga florida* was closely related to *Epacanthaclisis banksi* with high bootstrap values. Ascalaphidae, Myrmeleontidae and Nymphidae were cluster, so Myrmeleontiformia was confirmed as a monophyletic group (Aspöck et al. 2001; Cameron et al. 2009).

**Figure 1.**
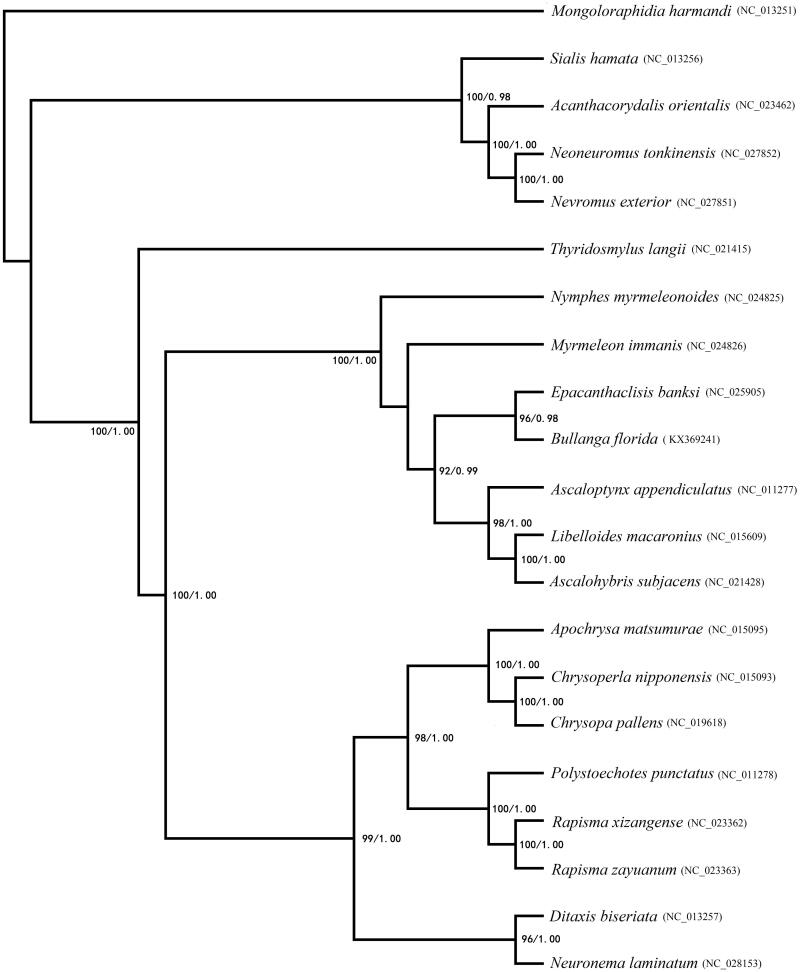
Phylogenetic relationships among Neuroptera families based on 13 mitochondrial protein-coding genes and constructed phylogenetic tree by BI and ML methods. Numbers on each node indicated the bootstrap value. Leaf names were presented as species names and Genbank accession number.

## Nucleotide sequence accession number

The complete genome sequence of *Bullanga florida* has been assigned GenBank accession number KX369241.
